# Supplementary material for: Two-decade trends and factors associated with overweight and obesity among young adults in Nepal
Source: PLOS Glob Public Health. 2023 Oct 31;3(10):e0002522. doi: 10.1371/journal.pgph.0002522 (PMC10617688; doi:10.1371/journal.pgph.0002522)
Supplement: S1 Fig — Trends of overweight and obesity among women and men from 1996 to 2019 using the WHO cutoffs and (b) Asian cutoffs. (DOCX) [file pgph.0002522.s001.docx]

1. **(b)**

**(c)**

**(d)**

**S1 Fig (a).** Trends in the prevalence of overweight and obesity among women of 18-29 years from 1996 to 2019 using the WHO cutoffs and **S1 (b)** Asian cutoffs, weighted percentages **S1 (c)** Trends in the prevalence of overweight and obesity among men of 18-29 years from 2007 to 2019 using the WHO cutoffs and **S1 (d)** Asian cutoffs, weighted percentages.
