# Supplementary material for: Two-decade trends and factors associated with overweight and obesity among young adults in Nepal
Source: PLOS Glob Public Health. 2023 Oct 31;3(10):e0002522. doi: 10.1371/journal.pgph.0002522 (PMC10617688; doi:10.1371/journal.pgph.0002522)
Supplement: S2 Fig — (DOCX) [file pgph.0002522.s002.docx]

**S2 Fig. Trends of median waist-to-hip ratio and waist-to-height ratio**
