# Supplementary material for: Two-decade trends and factors associated with overweight and obesity among young adults in Nepal
Source: PLOS Glob Public Health. 2023 Oct 31;3(10):e0002522. doi: 10.1371/journal.pgph.0002522 (PMC10617688; doi:10.1371/journal.pgph.0002522)
Supplement: S1 Table — (DOCX) [file pgph.0002522.s003.docx]

**S1 Table.** Demographic characteristics of women by years of survey

| **Background characteristics** |  | **Years of survey** | | | | | | |
| --- | --- | --- | --- | --- | --- | --- | --- | --- |
|  | **1996**  **n=3305 (%)** | **2001**  **n=3352 (%)** | **2006**  **n=4203 (%)** | **2007**  **n=654* (%)** | **2011**  **n=2492 (%)** | **2012**  **n=596* (%^)** | **2016**  **n=2467 (%)** | **2019**  **n=834* (%^)** |
| **Age in years** |  |  |  |  |  |  |  |  |
| Less than 20 | 447  (13.5) | 424  (12.7) | 826 (19.7) | 107 (21.0) | 468  (18.8) | 57 (12.1) | 457  (18.5) | 77 (12.7) |
| 20-24 | 1412  (42.7) | 1453  (43.3) | 1794 (42.7) | 269 (53.9) | 1079  (43.3) | 220 (40.5) | 1063  (43.1) | 325 (36.9) |
| 25-29 | 1446  (43.8) | 1475  (44.0) | 1583 (37.6) | 278 (25.1) | 945  (37.9) | 319 (47.4) | 947  (38.4) | 432 (50.4) |
| Mean age | 23.70  (±3.32) | 23.69  (±3.30) | 23.18  (±3.44) | 23.47 (±3.44) | 23.20  (±3.45) | 23.85 (±3.31) | 23.21  (±3.46) | 24.21  (±3.16) |
| **Residence** |  |  |  |  |  |  |  |  |
| Urban | 291  (8.8) | 323  (9.6) | 738 (17.0) | 296 (17.5) | 378  (15.2) | 102 (17.1) | 1552  (62.9) | 87 (8.1) |
| Rural | 3014  (91.2) | 3028  (90.4) | 3614 (83.0) | 358 (82.5) | 2114  (84.8) | 494 (82.9) | 915  (37.1) | 747 (91.9) |
| **Educational level** |  |  |  |  |  |  |  |  |
| No education/preschool | 2369  (71.7) | 2122  (63.3) | 1730 (39.8) | 177  (29.7) | 672  (27.0) | 219 (33.5) | 436  (17.7) | 206 (22.3) |
| Primary | 457  (13.8) | 557  (16.6) | 865 (19.9) | 217 (40.4) | 474  (19.0) | 89  (14.9) | 407  (16.5) | 194 (22.7) |
| Secondary | 411  (12.4) | 612  (18.2) | 1400 (32.2) | 134 (22.0) | 1018  (40.8) | 159 (27.6) | 990  (40.1) | 247 (27.9) |
| Higher | 69  (2.1) | 60  (1.8) | 356  (8.2) | 126 (8.0) | 328  (13.2) | 129 (24.1) | 634  (25.7) | 187 (27.1) |

*****unweighted frequencies; weighted total population size (N_1_) for 2007 survey=974268, N_2_ for 2012=3226203, N_3_ for 2019=3372911, **^**weighted percentage
