# Supplementary material for: Two-decade trends and factors associated with overweight and obesity among young adults in Nepal
Source: PLOS Glob Public Health. 2023 Oct 31;3(10):e0002522. doi: 10.1371/journal.pgph.0002522 (PMC10617688; doi:10.1371/journal.pgph.0002522)
Supplement: S2 Table — (DOCX) [file pgph.0002522.s004.docx]

**S2 Table.** Demographic characteristics of men by years of survey

| **Background characteristics** | **Years of survey** | | | |
| --- | --- | --- | --- | --- |
|  | **2007**  **n=541* (%)** | **2012**  **n=239***  **(%)** | **2016**  **n=1525 (%)** | **2019**  **n=362* (%^)** |
| **Age in years** |  |  |  |  |
| Less than 20 | 113  (22.0) | 32  (12.6) | 378 (24.7) | 50 (14.4) |
| 20-24 | 228  (44.6) | 96  (41.8) | 626  (41.0) | 137 (39.1) |
| 25-29 | 200  (33.4) | 111  (45.6) | 522 (34.2) | 175 (46.6) |
| Mean age | 22.95 (±3.46) | 23.87  (±3.47) | 22.74  (±3.47) | 23.89  (±3.40) |
| **Residence** |  |  |  |  |
| Urban | 287  (21.3) | 56  (23.9) | 1023  (67.1) | 54  (9.8) |
| Rural | 254  (78.7) | 183  (76.1) | 502 (32.9) | 308 (90.2) |
| **Educational level** |  |  |  |  |
| No education/preschool | 45  (16.8) | 24  (8.3) | 69  (4.6) | 59 (16.5) |
| Primary | 174  (41.0) | 46  (18.0) | 233 (15.3) | 61 (18.9) |
| Secondary | 119  (20.5) | 79  (35.8) | 695  (45.5) | 132 (41.6) |
| Higher | 203  (21.5) | 90  (37.8) | 528  (34.6) | 110 (23.0) |

*****unweighted frequencies; weighted total population size (N_1_) for 2007 survey=1148561, N_2_ for 2012=3014938, N_3_ for 2019=2867865, **^**weighted percentage
