# Supplementary material for: Two-decade trends and factors associated with overweight and obesity among young adults in Nepal
Source: PLOS Glob Public Health. 2023 Oct 31;3(10):e0002522. doi: 10.1371/journal.pgph.0002522 (PMC10617688; doi:10.1371/journal.pgph.0002522)
Supplement: Attachment — Submitted filename: Response to Reviewers.docx [file pgph.0002522.s006.docx]

To

Zulkarnain Jaafar

Academic Editor

PLOS Global Public Health

Dear Editor,

**RE: PGPH-D-23-00683- “Two-decade trends and factors associated with overweight and obesity among young adults in Nepal”**

Thank you very much for your decision and all the valuable comments and suggestions from you and the reviewers. We are heartily thankful for this opportunity to revise and resubmit the revision. We have attempted to address all the comments in the manuscript and provided a point-by-point response to each comment.

Please find attached the following for your kind consideration:

1) A rebuttal letter that responds to each comment of the reviewers
2) A marked-up copy of the manuscript highlighting changes as per the reviewers’ comments.
3) A clean version of the revised manuscript without track changes

We are really grateful to the editor and reviewers for helping us take the manuscript to this stage. We believe that the changes made have addressed all your comments and suggestions, and look forward to your kind acceptance for publication.

Yours Sincerely,

Sujata Shakya

Corresponding Author

**POINT BY POINT RESPONSES TO THE COMMENTS OF THE EDITOR AND REVIEWERS**

**PGPH-D-23-00683**

**Response to the Editor:**

**Journal Requirements:**

**1. Please upload a copy of Figure 3b which you refer to in your text on page 10. Or, if the figure is no longer to be included as part of the submission please remove all reference to it within the text.**

Response: Thank you for noticing this. We apologize for the mistake which occurred while revising the figure numbers and order. We have now corrected the figure number from 3b to 1b (line 263, page 13) in the revised manuscript.

**2. In the online submission form, you indicated that "Data are already open for public use upon permission". All PLOS journals now require all data underlying the findings described in their manuscript to be freely available to other researchers, either 1. In a public repository, 2. Within the manuscript itself, or 3. Uploaded as supplementary information.
This policy applies to all data except where public deposition would breach compliance with the protocol approved by your research ethics board. If your data cannot be made publicly available for ethical or legal reasons (e.g., public availability would compromise patient privacy), please explain your reasons by return email and your exemption request will be escalated to the editor for approval. Your exemption request will be handled independently and will not hold up the peer review process, but will need to be resolved should your manuscript be accepted for publication. One of the Editorial team will then be in touch if there are any issues.**

Response: We thank the editor for this comment. The data are already open and are available freely to anyone through DHS program site upon permission, which we mentioned in the Data Availability Statement and in the statistical analysis section line 212. And we have now added the citation to access the data under statistical analysis section, line 215.

**Response to the Reviewer #1:**

**Thanks for invitation of this good manuscript
the article is well written with good analysis and the topic is important , please you can only add paragraph for recommendation based on you findings' and dissemination plan of your results**

Response: We thank the reviewer for these remarkable comments and compliment. We had previously included the recommendations under the section “Implications of the Study”. Now we have revised the section as “Recommendations” in line 433. We have added a line mentioning about the dissemination plan to policy-makers and stakeholders in lines 446-447 of the track changed version.

*“The study findings will be shared with the concerned stakeholders and policy-makers for codevelopment of intervention strategies to prevent overweight and obesity.”*

**Response to the Reviewer #2:**

**-general comment: many weak sentence structures; avoid long sentences**

Response: We agree that there were many weak sentence structures and sentence errors. We have now rephrased the long sentences and made them concise. We have also thoroughly revised our manuscript and also got the language editing done by the American Journal of Experts – all changes are tracked in the manuscript. We hope that now the errors are none or minimal.

**- it was mentioned that "Young adulthood of age 18 to 25 years, is a critical stage.." but it is not explained why you included the target group till 29 years old?**

Response: Thanks for highlighting this important concern. We have now included the explanation about the controversy of the exact age range for considering young adulthood in line numbers 88-92. In the revised text (lines 118-119), we have explained why we included the age range 18-29 years.

*“We used data only of young adults since our concern was the overweight and obesity in this group. We considered the young adults aged 18-29 years, similar to a previous study conducted in Nepal [29].”*

**- some sentences are missing in- text citation.**

Response: We have realized missing in-text citations in some areas. Now we have added the citation in lines 101, 120 and 157.

**- It was mentioned "There are inadequate evidences in Nepal to explain the current trends of overweight and obesity among young adults group and the socio-economic factors that are underway. There are only scattered findings from small scale studies through which it is challenging to draw concrete conclusion." but you already mentioned above that "Different studies in Nepal showed high prevalence among the young adults group [15,17,25]". Contradictory statements.**

Response: We thank the reviewer for this comment. By the above statement, we meant to highlight the paucity of information on the trend study on overweight and obesity specifically among young adults. The available few studies are small scale cross-sectional studies assessing the existing prevalence and not the long-term trends. We have now clarified this in lines 96, and 99-102.

*“There is inadequate evidence in Nepal to explain the current trends of overweight and obesity among young adults and the socioeconomic factors that affect them. There are few findings from small- scale cross-sectional studies [15,17,26,27] through which it is challenging to draw concrete conclusions regarding the current trends.”*

**The study design is not clear enough. It was mentioned " We used several cross-sectional surveys.." Do you mean you used the data collected previously from these surveys? You did not do primary data but used data collected previously?**

Response: Thanks a lot to the reviewer for pointing out this. We did not conduct primary data collection but used the data from national-level cross-sectional surveys collected previously. We have rephrased the words in lines 114-115 for clarity.

*“We used data from several cross-sectional surveys conducted among nationally representative adult samples of from 1996-2019.”*

**- "The total participants enrolling in the surveys in different years were 21,281." Is this number related to the age group 18-29 only?**

Response: Yes, this number is related to the age group 18-29 years only, which we have now mentioned in line 120-121. We thank the reviewer for highlighting this.

*“The total number of participants aged 18-29 years enrolled in the DHS and WHO STEPS surveys in different years was 21,281.”*

**- in the WHO STEPS Surveys: "The STEPS surveys are population-based surveys among adults aged 15–69 years conducted using internationally comparable, standardized and integrated surveillance tool.." but you took data from the age group 18-29 only? please explain**

Response: We thank the reviewer for this concern. It is correct that the STEPS surveys are conducted among adults aged 15-69 years and the DHS surveys are conducted among adults aged 15-49 years. But we extracted data only from the age group 18-29 years since our research concern only young adults. We have provided some texts in the methods section lines 118-120 the reason behind including the age group 18-29 years for our study.

*“We used data only from young adults since our concern was the overweight and obesity among this group. We considered young adults aged 18-29 years, similar to a previous study conducted in Nepal [29].”*

**- in the DHS surveys you stated that “We utilized data from five surveys between 1996 and 2016.” was it continuously? or for a few years only? please explain**

Response: Thank you so much for pointing this out. We have now specified in lines 124-126 that we used data from five consecutive surveys from 1996 to 2016 which were conducted every 5 years.

*“We utilized data on overweight and obesity based on BMI and related independent variables from five consecutive surveys between 1996 and 2016.”*

**-What type of data did you collect from each of these surveys? This should be clearly stated.**

Response: We have now specified in the methods section, lines 124-126 and lines 147-149 what type of data we collected from the DHS surveys and the STEPS surveys.

*“We utilized data on overweight and obesity based on BMI and related independent variables from five consecutive surveys between 1996 and 2016.”*

*“From these surveys, we accessed data on overweight and obesity based on BMI, waist-to-hip ratio and waist-to-height ratio, and the other sociodemographic factors.”*

**- ethical consideration: did you take IRB? Did you use identifiable data from the two surveys?**

Response: Since we used secondary data from the surveys which were already approved by the national ethical board, we did not again apply for ethical approval. There were no identifiable data in the two surveys as mentioned in the approval letter. We have mentioned these under the ethical considerations section, in lines 155-161.

*“The DHS and WHO STEPS surveys were approved by the Nepal Health Research Council (NHRC) Ethical Review Board. Informed consent for the surveys was obtained from all the participants who were interviewed [30–32]. In this study, a separate approval from the ethical board was not required.”*

*“There are no names of individuals, household addresses or any identifiable data in the data files.”*

**- it was stated “We found very limited studies on trends of overweight/obesity prevalence, especially among the young adults to compare with our findings, as most of the studies were conducted among reproductive-age (15- 49 years) women.” But your age group (18-29) is included in this group so why is this a limitation?**

Response: Thank you for this concern. We meant by this that there are a few studies conducted specifically focusing on young adults. We agree that the above sentence created confusion. So, we have removed the second part of the sentence in lines 356-357.

**- it was stated, “The DHS surveys are targeted to estimate the basic demographic and health indicators and focus mainly on maternal and child health issues.” How is maternal and child health related to your research and target group (18-29)?**

Response: We apologize for this confusion. The DHS surveys obtain information on both general health indicators as well as maternal and child health indicators. These surveys elaborately collect information on maternal and child health issues but include other general health information too including communicable and non-communicable diseases, health practices, lifestyles etc. We have revised the sentence in line 417-419 for clarity.

“*The DHSs are targeted to estimate the basic demographic and health indicators and focus mainly on general health issues including maternal and child health.”*

**- “Our study results indicate that the highest risk groups for increasing overweight and obesity prevalence are the age-group 25-29 years,” how did you conclude this?? Among which risk groups?**

Response: Since the prevalence of overweight and obesity was highest in the age group 25-29 years with the rapidly increasing trend, we highlighted this age group as the risk group. We have now revised the sentence structure for more clarity in lines 437-440.

“*Our study results indicated that the upward trends in the prevalence of overweight and obesity were greatest in the 25-29 age group, which calls for urgent action towards the implementation of obesity control interventions targeting these risk groups at the national level.”*
