# Supplementary material for: Two-decade trends and factors associated with overweight and obesity among young adults in Nepal
Source: PLOS Glob Public Health. 2023 Oct 31;3(10):e0002522. doi: 10.1371/journal.pgph.0002522 (PMC10617688; doi:10.1371/journal.pgph.0002522)
Supplement: Attachment — Submitted filename: Response to Reviewers_R2.docx [file pgph.0002522.s008.docx]

To

Zulkarnain Jaafar

Academic Editor

PLOS Global Public Health

Dear Editor,

**RE: PGPH-D-23-00683- “Two-decade trends and factors associated with overweight and obesity among young adults in Nepal”**

Thank you so much for your response and all the valuable suggestions received from you and the reviewer. We are really grateful and happy for this opportunity to revise and resubmit the revision. We have revised our manuscript and tried to address all the comments provided. As suggested, we also uploaded all the figure files to the Preflight Analysis and Conversion Engine (PACE) digital diagnostic tool.

Please find attached the following documents as required for your kind consideration:

1) A rebuttal letter that responds to each comment of the reviewers
2) A marked-up copy of the manuscript highlighting changes as per the reviewers’ comments.
3) A clean version of the revised manuscript without track changes

We believe that the revisions made have now addressed all the comments given by the editor and the reviewer. Please let us know if there are any more edits necessary. We are happy to follow your suggestions. We look forward to your kind acceptance for publication.

Yours Sincerely,

Sujata Shakya

Corresponding Author

**POINT BY POINT RESPONSES TO THE COMMENTS OF THE EDITOR AND REVIEWERS**

**PGPH-D-23-00683R1**

**Response to the Editor:**

**Journal Requirements:**

**Please review your reference list to ensure that it is complete and correct. If you have cited papers that have been retracted, please include the rationale for doing so in the manuscript text, or remove these references and replace them with relevant current references. Any changes to the reference list should be mentioned in the rebuttal letter that accompanies your revised manuscript. If you need to cite a retracted article, indicate the article’s retracted status in the References list and also include a citation and full reference for the retraction notice.**

**Response:** Thank you for the information on the requirements of referencing. We have reviewed the reference to check for any errors or incompleteness. We found few mistakes in the references, which we corrected in the revised version. The corrections which we made are listed below:

1. Current Reference number 14, Khan & Rasheed 2018: There was an extra word “introduction” at the end of the article title which we deleted.
2. Current Reference number 18, Balarajan & Villamore, 2009: This reference was repeated in reference number 27 which we deleted. We also edited the citation number of this article in line numbers 92 and 331.
3. We removed the web page address from the journal articles having DOI.

With regards to checking for retraction of the articles we cited, we checked each article in the journal site as well as checked for any retractions by using “Retraction watch database”. We did not find any retracted articles in our reference list.

**Response to the Reviewer #3**

**This manuscript presents an important investigation of overweight and obesity in youth, measured over two decades period. The combination of information sources and the application of various analysis methodologies constitute good support for the information obtained. The analysis is complete and indicates the scope and limitations of the data of the trends described as a result of the research. The discussion of the results is pertinent and is aimed at decision makers regarding this public health problem. The conclusions are correctly written and are consistent with the objectives. However, I would like to make two writing suggestions that may improve it:**

**In section Discussion**

**Sentence: "Older age, female sex and married status increased the likelihood of overweight, whereas older age, female sex and urban residence increased the likelihood for obesity."**

**I suggest: The likelihood of overweight increased in groups of older age, female sex and married status, whereas the likelihood of obesity was increased in groups of older age, female sex and urban residence.**

**Response:** We heartily thank the reviewer for the above remarks, and for the suggested texts. We have now deleted the earlier sentence and replaced with the suggested one in lines 292-293.

**In section Predictors/risk factors for overweight and obesity**

**Sentence: "However, male obesity was found to be more common than female obesity in Asian and North African countries [37]. This was linked to the social disadvantages for women leading to less food intake by women and more intake by men; however, this was not empirically confirmed."**

**I suggest: However, male obesity was found to be more common than female obesity in Asian and North African countries [37]. That finding wanted to be explained with the hypothesis: the social disadvantages for women leading to less food intake by women and more intake by men; however, it could not be verified empirically.**

**Response:** Thank you so much for your suggestion. We have now included the suggested sentences with very few changes in lines 335-338. I believe these are fine.

*“However, male obesity was more prevalent than female obesity in Asian and North African countries [37]. This observation prompted an exploration of a hypothesis: whether social disadvantages for women result in reduced food intake by women and increased intake by men. Nevertheless, empirical verification of this hypothesis was inconclusive.”*
